# Adaptive optics, microscopy, and beyond: an interview with Professor Na Ji

**DOI:** 10.1117/1.NPh.11.2.020401

**Published:** 2024-04-15

**Authors:** Anderson Chen

**Affiliations:** Prisma Therapeutics, Inc., Cambridge, Massachusetts, United States

## Abstract

Prof. Na Ji (UC Berkeley) discusses her pioneering work and motivation in adaptive optics, microscopy, and beyond, in an interview with former trainee Anderson Chen (Prisma Therapeutics, Inc.).

**Figure:**
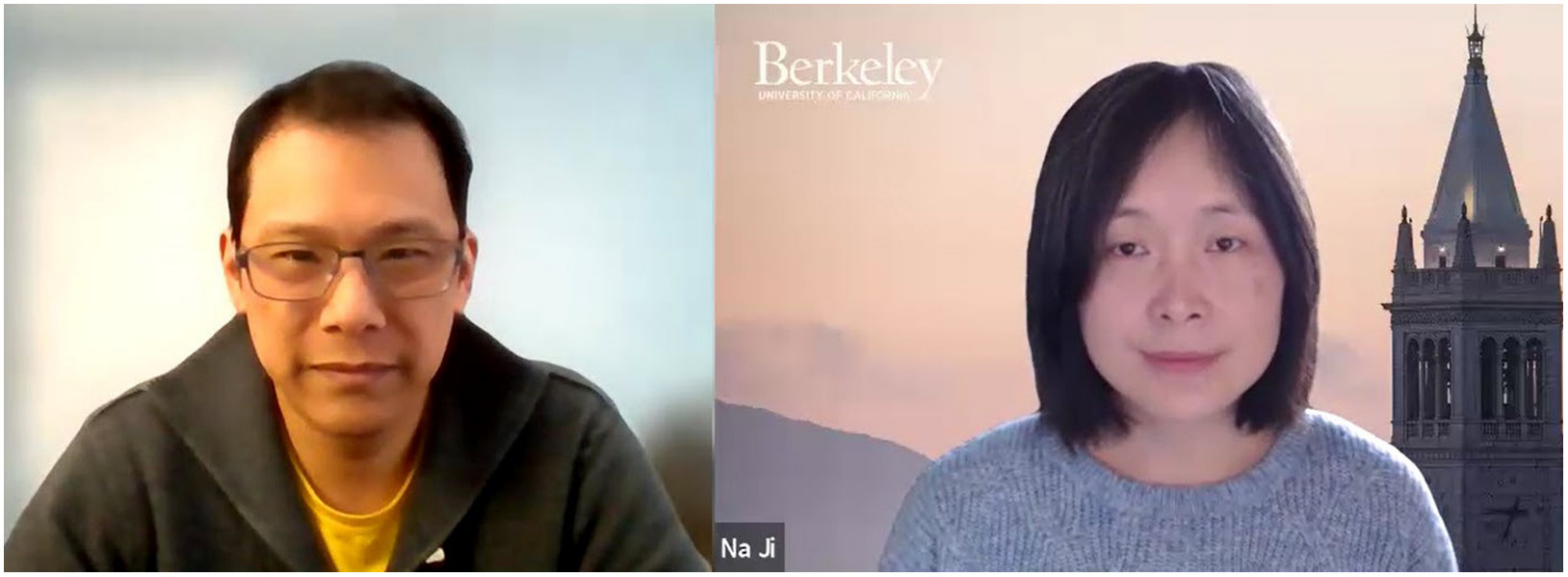
(Right) Anderson Chen (Prisma Therapeutics, Inc.) interviews Prof. Na Ji (UC Berkeley) about her pioneering work in adaptive optics and microscopy for brain imaging. View a video recording of the interview at https://doi.org/10.1117/1.NPh.11.2.020401.

